# Connective tissue growth factor mediates transforming growth factor β-induced collagen expression in human endometrial stromal cells

**DOI:** 10.1371/journal.pone.0210765

**Published:** 2019-01-29

**Authors:** Mei-Leng Cheong, Tsung-Hsuan Lai, Wen-Bin Wu

**Affiliations:** 1 Department of Obstetrics and Gynecology, Cathay General Hospital, Taipei, Taiwan; 2 Department of Obstetrics and Gynecology, School of Medicine, College of Medicine, Taipei Medical University, Taipei City, Taiwan; 3 School of Medicine, Fu-Jen Catholic University, New Taipei City, Taiwan; 4 Graduate Institute of Biomedical and Pharmaceutical Science, Fu-Jen Catholic University, New Taipei City, Taiwan; Chang Gung University, TAIWAN

## Abstract

**Background:**

Adenomyosis is a medical condition defined by the abnormal presence of endometrial tissue within the myometrium, in which fibrosis occurs with new collagen deposition and myofibroblast differentiation. In this study, the effect of several mediators and growth factors on collagen expression was investigated on human endometrial stromal cells (fibroblasts) derived from adenomyotic endometrium.

**Experimental approach:**

RT-PCR, Western blot analysis, pharmacological interventions and siRNA interference were applied to primary cultured human endometrial stromal cells (fibroblasts). Immunohistochemistry was used to analyze protein expression in adenomyotic endometrium tissue specimens.

**Results:**

Of the tested mediators, transforming growth factor β1 (TGFβ1) and its isoforms were effective to induce collagen and connective tissue growth factor (CTGF) expression. Collagen and CTGF induction by TGFβ1 could be reduced by the inhibitors targeting DNA transcription, protein translation, and Smad2/3 signaling. Interestingly, TGFβ1 induced Smad2/3 phosphorylation and CTGF mRNA expression, but not collagen mRNA expression, suggesting that TGFβ1 mediates collagen expression through CTGF induction and Smad2/3 activation. In parallel, TGFβ1 and CTGF also induced expression of heat shock protein (HSP) 47, a protein required for the synthesis of several types of collagens. However, only CTGF siRNA knockdown, could compromise TGFβ1-induced collagen expression. Finally, the immunohistochemistry revealed vimentin- and α-SMA-positive staining for (myo)fibroblasts, TGFβ1, collagen, and CTGF in the subepithelial stroma region of human adenomyotic endometria.

**Conclusion and implications:**

We reveal here that TGFβ1, collagen, and CTGF are expressed in the stroma of adenomyotic endometria and demonstrate that TGFβ1 can induce collagen production in endometrium-derived fibroblasts through cellular Smad2/3-dependent signaling pathway and CTGF expression, suggesting that endometrial TGFβ may take part in the pathogenesis of adenomyosis and ectopic endometrium may participate in uterine adenomyosis.

## Introduction

Uterine adenomyosis is a medical condition defined by the abnormal presence of endometrial tissue within the myometrium and the main mechanisms include sex hormone aberrations, inflammation and neuroangiogenesis, proliferation and fibrosis [1]. However, the exact etiology of adenomyosis remains unclear. Recently, by means of magnetic resonance imaging technology, it was reported that uterine adenomyosis can be further classified into four subtypes based on their localizations and all types usually have an aspect of fibrosis [2, 3].

Tissue fibrosis generally results from remodeling, which is a critical aspect of wound repair in all organs. Characteristically, fibrosis includes the activation of stromal fibroblasts within connective tissue, namely myofibroblasts with expression of α-smooth muscle actin (α-SMA). The α-SMA can be organized into contractile microfilaments [4]. In addition, the formation of fibrosis correlates with extracellular matrix (ECM) production, new collagen deposition, and transforming growth factor β (TGFβ)-induced myofibroblast differentiation [5, 6]. For example, TGFβ can switch vascular smooth muscle cells (VSMCs) from a contractile to a proliferative synthetic phenotype at sites of vascular injury [7, 8]. Recent evidence also suggested that TGFβ1 plays a central role in the initiation of chronic rhinosinusitis (CRS) without nasal polyp and participates in inflammation and remodeling patterns in early stage of CRS [9].

Connective tissue growth factor (CTGF) is a secreted protein, belonging to a member of the CCN family of matricellular proteins [10]. The CTGF function *in vivo* has mainly focused on its role as a central mediator of tissue remodeling and fibrosis, including excess ECM synthesis in multiple fibrotic diseases [11]. In addition to CTGF, heat shock protein 47 (HSP47) is a stress-related protein with molecular weight of 47-kDa, which is mainly localized to the endoplasmic reticulum of cells for synthesizing collagens. It is a human chaperone protein for collagens which folds the procollagens into their appropriate protein conformations [12]. HSP47 has been shown to regulate ECM accumulation in renal proximal tubular cells induced by TGFβ1 through MAPK-related pathways [13].

Ectopic and eutopic endometrium in adenomyosis undergo cyclic or repeated tissue injury and repair [14, 15] and may cause fibrosis. Meanwhile, it has been reported that integrin α2/α3β1 and E-cadherin significantly increase during the menstrual cycle in both of the endometriotic and adenomyotic endometria [16]. The ligands for integrin α3β1 include fibronectin, laminin, and collagen [17]. Interestingly, an increase in collagen content has also been reported in adenomyosis [18, 19].

Recently, the abundant and persistent myofibroblasts expressing α-SMA/type I collagen were shown to be observed at endometrial-myometrial junctional zone (EMJZ) in adenomyotic uteri [20]. In parallel, staining of markers of epithelial-mesenchymal transition (EMT) and fibroblast-to-myofibroblast transdifferentiation (FMT) become more progressively marked when adenomyosis proceeded, along with an increase in TGFβ1 and Smad3 phosphorylation, leading to increased tissue fibrosis in adenomyotic lesions [21]. Fibroblasts are usually recruited to the site of injury and undergo TGFβ-mediated fibroblasts transdifferentiation into myofibroblasts [20]. Therefore, this study was sought to investigate the possible role of endometrial TGFβ and stromal cells contribute to the pathogenesis of adenomyosis. The relationship between TGFβ, CTGF, HSP47 and collagen expression was explored in human endometrial stromal cells (HESCs, stromal fibroblasts) derived from human adenomyotic endometrium and their expressions were also examined in adenomyotic endometrium specimens.

## Materials and methods

### Materials

Human EGF and bFGF were from Thermo Fisher Scientific (NY, USA). Human TGFβ1 and TNF-α were from R&D systems, Inc. (MN, USA). Human TGFβ2 and TGFβ3 were from Prospec-Tany TechnoGene Ltd. (East Brunswick, NJ, USA). Thrombin, PD98059, SB202190, SP600125 were purchased from Sigma-Aldrich Chemical Co. (St Louis, MO, USA). Human recombinant VEGF was purchased from Prospect Biotech (Rehovot, Israel). Estrogen and progesterone were purchased from Cayman Chemical Co. (Ann Arbor, Michigan, USA). Recombinant human CTGF was obtained from Thermo Fisher Scientific eBioscience (Waltham, MA, USA). The antibodies (Abs) raised against vimentin (sc-6260), HSP47 (sc-8352) and phospho-Smad2/3 (sc-11769) were from Santa Cruz Biotechnology Inc. (Dallas, TX, USA). The Ab for total Smad2/3 (#3102) was purchased from Cell Signaling Technology, Inc. (Danvers, Massachusetts, USA). The Ab for α-SMA (GTX100034) was purchased from GeneTex (Hsinchu City, Taiwan). The Abs for collagen (ab34710), CTGF (ab6992) and α-tubulin (ab7291) were purchased from Abcam (Cambridge, MA). TGFβ isoforms were dissolved in 4 mM HCl/0.1% BSA (vehicle).

### Preparation of human endometrial stromal cells (HESCs)

This study has been approved by the Ethics Committee of the Cathay General Hospital (Taipei City, Taiwan) (Permission no: CGH-P102029) and conducted with the written informed consent to the patients. The endometrium samples were obtained at secretory phase of menstrual cycle via hysterectomy from 25 patients with adenomyosis and the HESCs were prepared from the endometrium of the uterus. The procedures of preparation of HESCs have been described previously with some modifications [22, 23]. Briefly, tissue fragments (minced endometrial tissue) were incubated with Dulbecco’s modified Eagle’s medium-F12 (DMEM-F12) containing collagenase (1 mg/ml) (Sigma-Aldrich Chemical Co.), penicillin (100 U/ml), streptomycin (100 μg/ml), and amphotericin B (25 μg/ml) (Thermo Fisher Scientific, NY, USA) at 37°C for 1 h with gentle agitation. After two times of centrifugation, the supernatant was transferred into a culture flask and allowed the cells to adhesion. The obtained cells were endometrial stromal fibroblasts, namely human endometrial stromal cells (HESCs). HESCs were cultured in DMEM-F12 containing above mentioned antibiotics, amphotericin B, and 10% fetal bovine serum (Thermo Fisher Scientific). The cells were identified and characterized by immunofluorescence staining for the positive staining of vimentin (Santa Cruz Biotechnology).

### Western blot analysis

The cell lysates were prepared as previously described [24]. The total proteins were analyzed by electrophoresis, blotted onto PVDF membranes, and then probed using an Ab raised against the indicated protein. The immunoblots were developed using Immobilon Western Chemiluminescent HRP Substrate (EMD Millipore, Billerica, MA, USA).

### RT-PCR analysis for mRNA expression

Oligonucleotide polymerase chain reaction (PCR) primers targeting human collagen, CTGF, HSP47, and β-actin were synthesized (Table 1). The total RNA extraction, reverse transcription of total RNA to cDNA, and PCR analysis have been previously described [24]. The PCR was performed with 30 cycles of denaturation at 94°C for 1 min, annealing at 52°C for 1 min, and elongation at 72°C for 1.5 min on the ABI 7200 Thermal Cycler (Applied Biosystems, Foster City, CA, USA). The PCR products were analyzed by 1–2% agarose gel electrophoresis.

**Table 1:** Primers for the reverse transcription polymerase chain reaction.

Gene	Forward primer (5’->3’)	Reverse primer (5’->3’)	Expected product size (bp)
*collagen*	CATCACCTACCACTGCAAGAAC	ACGTCGAAGCCGAATTCC	353
*CTGF*	CCAAGGACCAAACCGTGGT	TACTCCACAGAATTTAGCTCG	278
*HSP47*	GCGGGCTAAGAGTAGAATCG	ATGGCCAGGAAGTGGTTTG	154
*β-actin*	ATCATGTTTGAGACCTTCAA	CATCTCTTGCTCGAAGTCCA	314

### Determination of expression patterns of fibroblasts and protein expression in adenomyotic endometria

The expression patterns of stromal (myo)fibroblasts, TGFβ, collagen, and CTGF in adenomyotic endometria were determined through immunohistochemistry. The endometrium samples were obtained at secretory phase of menstrual cycle via hysterectomy from the patients with adenomyosis, which has also been approved by the Ethics Committee of the Cathay General Hospital (Taipei City, Taiwan) (Permission no: CGH-P102029). The patients had no hormone replacement therapy within 2 months, endometritis, endometrial polyp, pelvic inflammatory diseases, sex transmitted diseases, and any other pathological conditions in uterine cavity. Briefly, tissue sections were deparaffinized, and the slides were hydrated in graded ethanol before use. The sections were subsequently washed in Tris-buffered saline (TBS; 10 mM Tris-HCl, 150 mM NaCl, pH 7.4) containing 1% CaCl_2_, immersed in sodium citrate buffer (pH 6.0), and heated on a water bath for 20 min. The slides were incubated at 4°C overnight with the primary Ab specific for TGFβ1, type I collagen, and CTGF (Abcam, Cambridge, MA, USA) after being blocked with a TBS buffer containing 10% FBS. The slides were then washed with TBS, incubated with Super Enhancer and Poly-HRP, and then developed with one-step 3-amino-9-ethylcarbazole (AEC) using the Super Sensitive Polymer-HRP IHC Detection System (Biogenex Laboratories, Inc., Fremont, CA, USA) for 5–30 min. Sections were counterstained in hematoxylin for 20-40s, washed with tap water, and mounted with 100% glycerol.

### siRNA transfection

SiGenome non-targeting control siRNA pool (catalog #D-00126), human CTGF siRNA smart pool (RNA accessions: NM_001901.2, catalog #M-012633), and human HSP47 (serpin family H member 1; SERPINH1) siRNA SMARTpool (RNA accessions: NM_001207014.1, catalog #M-011230) were purchased from Dharmacon RNAi Technologies (Thermo Fisher Scientific). The transfection was performed according to the manufacturer’s protocol with minor modifications. The cell cultures were transfected with control or siRNAs (150 nM) for 72 h using the DharmaFECT transfection reagent (Thermo Fisher Scientific). The cells were collected and analyzed through Western blotting.

### Statistical analysis

Data were expressed as means ± standard error mean (SEM). Comparison of means of two groups of data was made by using the unpaired, two-tailed Student *t* test.

## Results

### Preparation of HESCs and screening of mediators affecting collagen and CTGF expression

**Fig 1:**
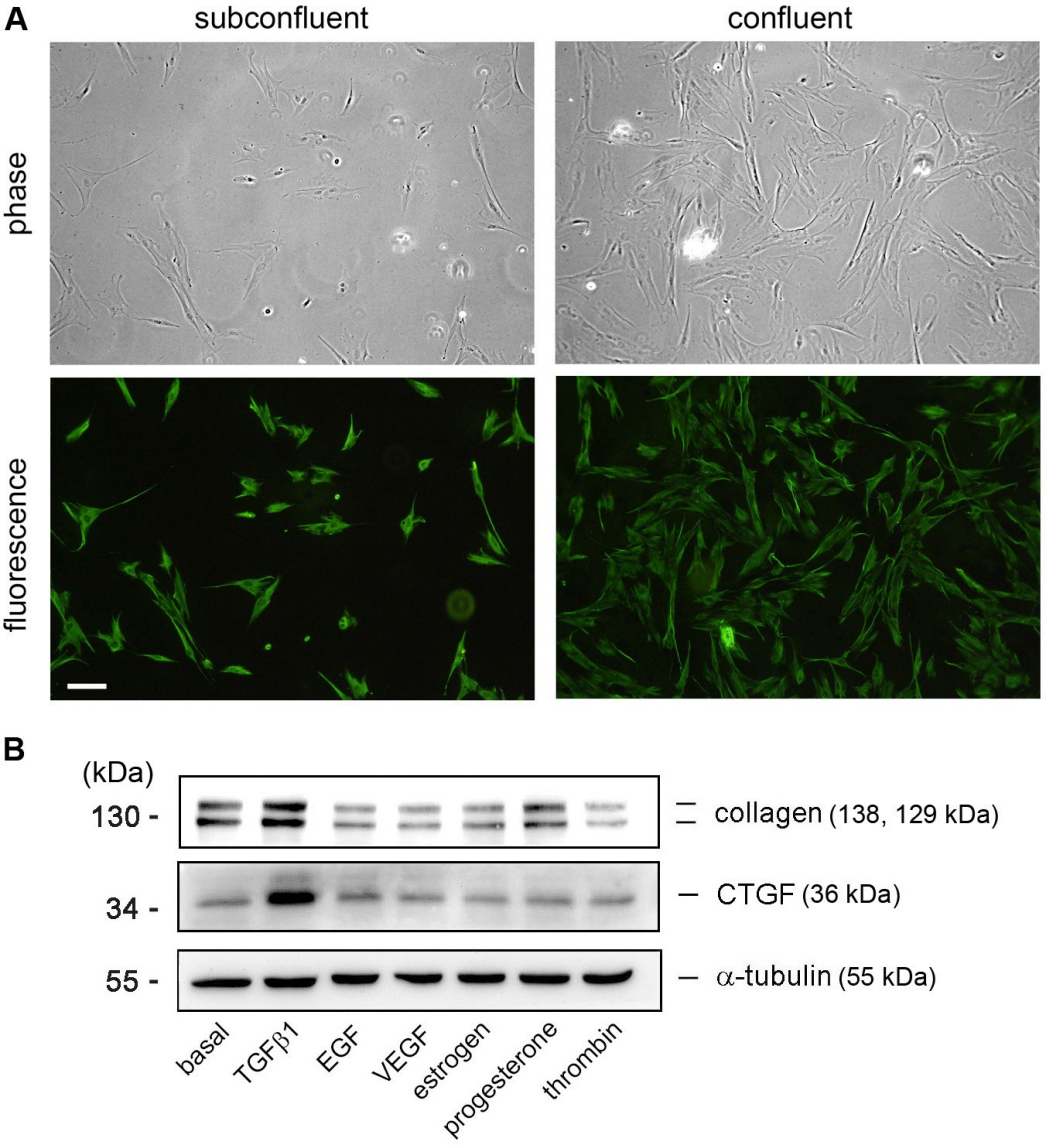
Characterization of prepared HESCs and effects of mediators on collagen and CTGF expression in HESCs. (A) HESCs were isolated and prepared as described in the Materials and Methods. Cell morphology was analyzed through phase-contrast microscopy (upper panels) and the fibroblast identity was determined through immunofluorescence microscopy (lower panels) (n = 3). (B) Effect of mediators on collagen and CTGF expression. Cells were treated with mediators for 16 h. The concentration for each mediator was TGFβ1 (10 ng/ml), EGF and VEGF (50 ng/ml), estrogen (1 μM), progesterone (2 μM), and thrombin (2 U/ml). The protein expression was determined through Western blotting (n = 2–3).

The HESCs were isolated and prepared from human endometrium with adenomyosis. The identity of obtained fibroblasts was confirmed by their morphology and by the presence of a fibroblast specific marker-vimentin. In Fig 1A, the subconfluent and confluent HESCs were analyzed through microscopy and their morphology was shown to be spindle-like (upper panels), as previously reported by others [22]. In addition, an immunofluorescence staining using an anti-vimentin Ab and FITC-conjugated 2^nd^ Ab shows that the cytoplasm of these cultured cells was with strong fluorescence reactivity (lower panels), indicating that they were fibroblasts.

To investigate which mediator(s) affect(s) collagen and CTGF expression in HESCs, the cells were stimulated with TGFβ1, EGF, VEGF, sex hormones (estrogen and progesterone), and thrombin. The cell lysates were analyzed through Western blotting. In Fig 1B, the collagen expression was in a certain amount (basal level) in the HESCs. While the addition of EGF, VEGF, estrogen, progesterone, and thrombin to cells did not induce collagen expression, stimulation of TGFβ1 markedly enhanced collagen expression and CTGF expression in the HESCs.

### Effects of TGFβ on collagen and CTGF protein and mRNA expression

**Fig 2:**
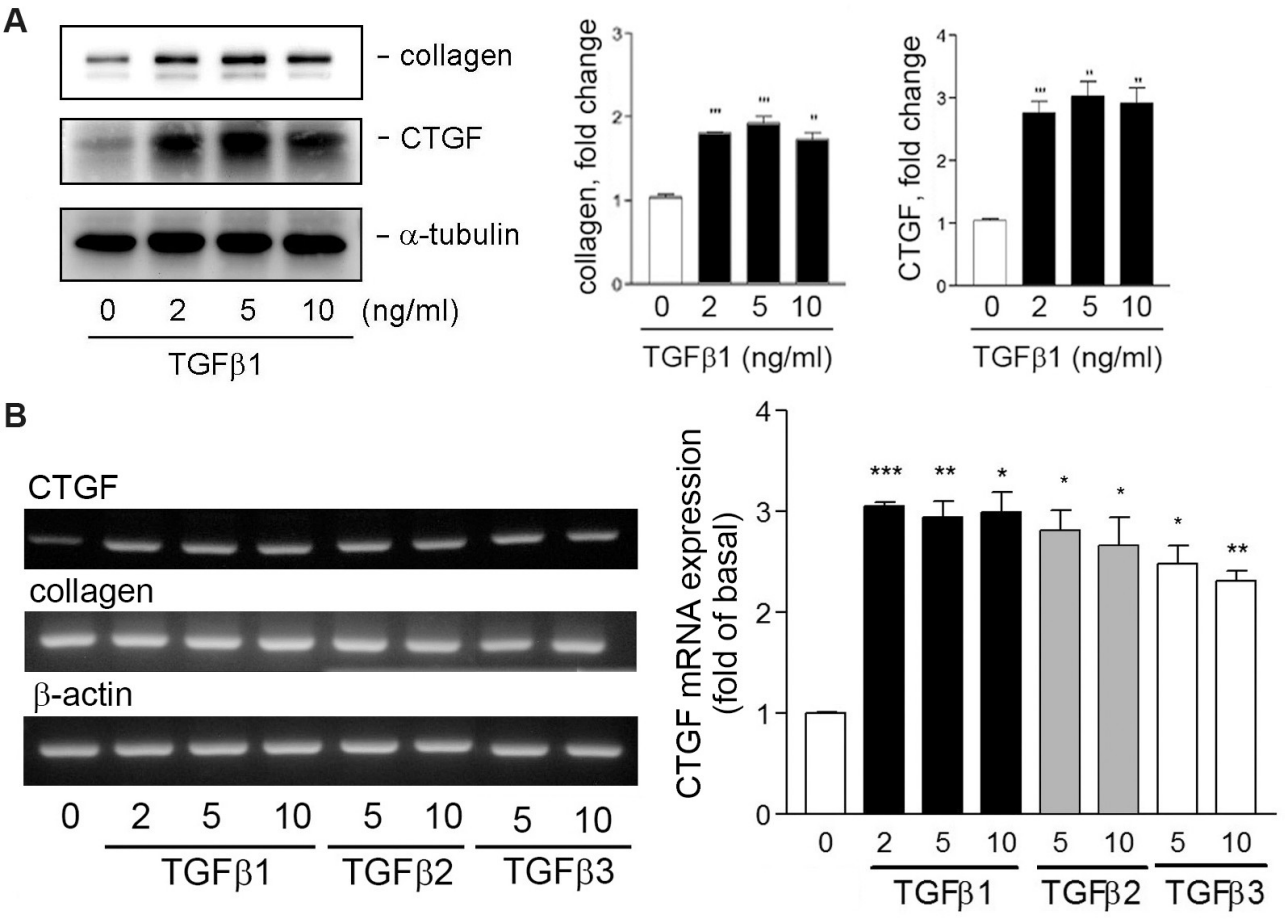
Effects of TGFβ isoforms on collagen and CTGF protein and mRNA expression in HESCs. (A) Cells were treated with the indicated concentration of TGFβ1 for 16 h and then analyzed through Western blotting (n = 3). (B) Effect of TGFβ isoforms on collagen and CTGF mRNA expression in HESCs. Cells were treated with vehicle or TGFβ isoforms (ng/ml) for 4 h. The mRNA expression level of collagen, CTGF, and β-actin was analyzed through RT-PCR and densitometry (n = 2). *P < 0.05, **P < 0.01, and ***P < 0.001 versus vehicle treatment only (basal).

Next, the concentration-dependent effects of TGFβ1 on collagen and CTGF expression were determined. As shown in Fig 2A, TGFβ1 stimulation induced an increase in collagen and CTGF expression, 2ng/ml TGFβ1 was sufficient to cause the expression of collagen and CTGF. In parallel, the effect of TGFβ1 on collagen and CTGF mRNA expression was explored through RT-PCR. Surprisingly, while the collagen mRNA expression was not affected, the CTGF mRNA was induced by TGFβ1 treatment (Fig 2B). We also tested whether the TGFβ isoforms have similar activity on HESCs in inducing collagen and CTGF mRNA expression. The TGFβ2 and β3 have similar but relatively weak activity compared to TGFβ1 on collagen and CTGF protein and mRNA expression (Fig 2B), this was possibly due to that TGFβ isoforms share with 70–80% of homologies in their amino acid sequences [25]. Taken together, we showed that TGFβ1 can induce collagen and CTGF expression in HESCs and its isoforms share similar activity on induction of those proteins.

### Effect of TGFβ1 on heat shock protein 47 (HSP47) expression

**Fig 3:**
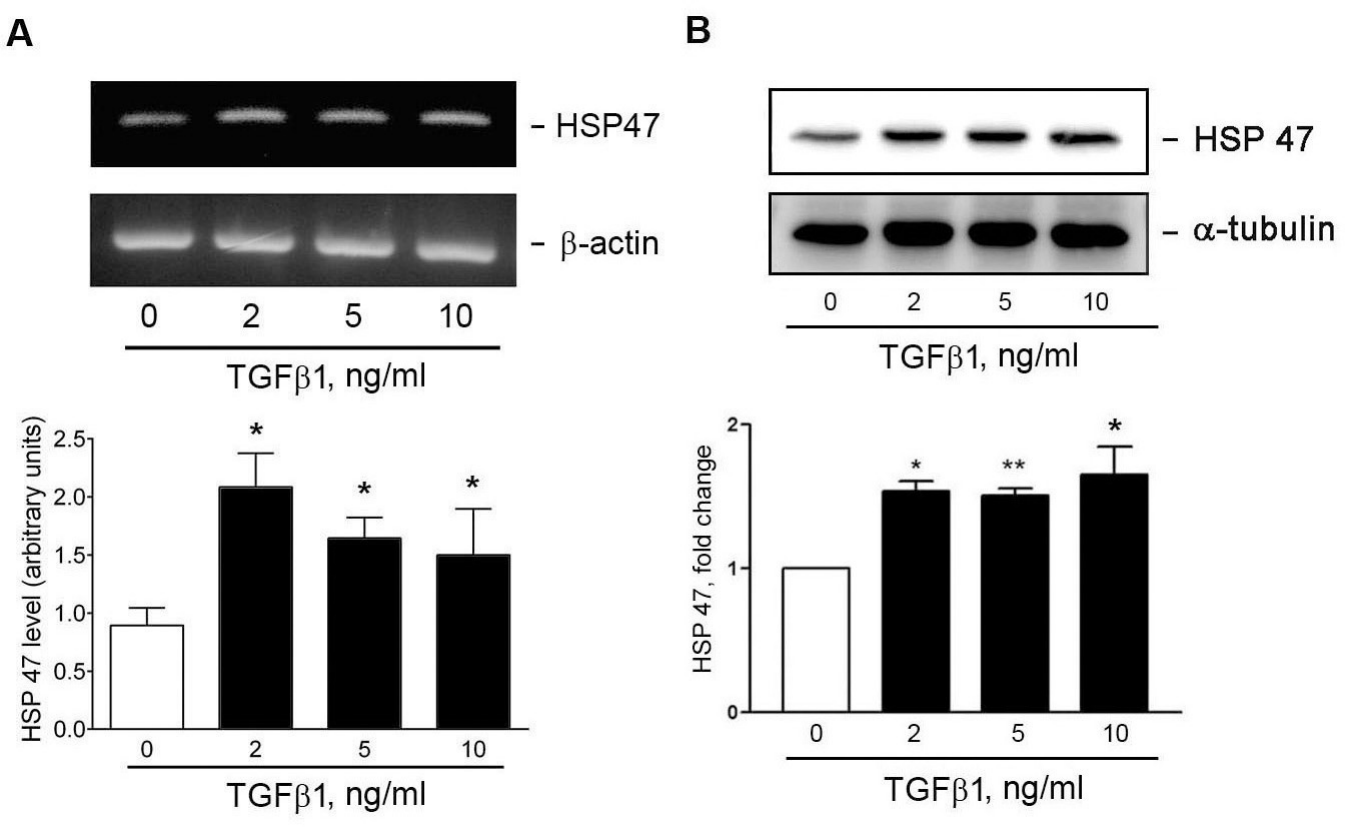
TGFβ1 induces HSP47 expression in HESCs. (A) Cells were treated with vehicle or TGFβ1 for (A) 4 h or (B) 16 h. The HSP47 expression was analyzed through (A) RT-PCR and (B) Western blotting (n = 3). *P < 0.05, and **P < 0.01 versus vehicle treatment only (basal).

We have shown that TGFβ1 could induce both of collagen and CTGF protein expression in the HESCs (Figs 1 and 2). Since HSP47 has been reported to mediate TGFβ1-induced ECM deposition in renal tubular cells [13], we examined whether TGFβ1 can induce HSP47 expression in HESCs. In Fig 3, treatment of cells with TGFβ1 led to HSP47 mRNA (panel A) and protein expression (panel B).

### CTGF enhances collagen and HSP47 expression

**Fig 4:**
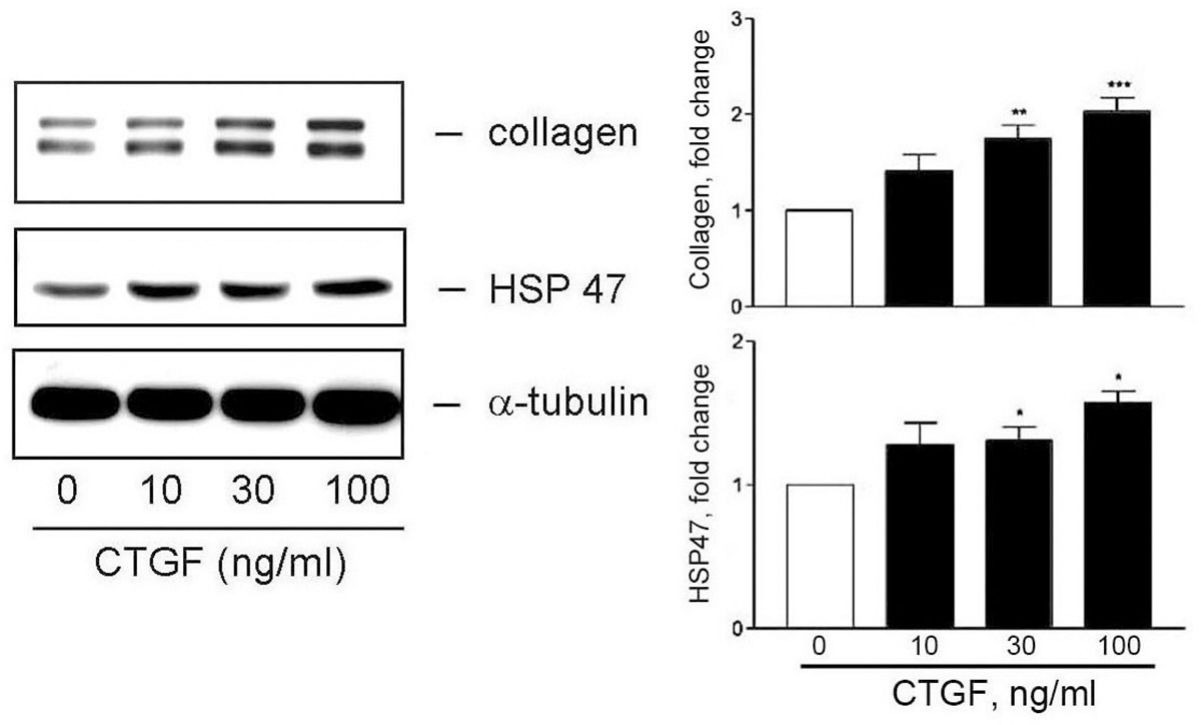
CTGF enhances collagen and HSP47 expression in HESCs. Human CTGF was added to cells for 16 h. The cells were collected and collagen and HSP47 expression were analyzed through Western blotting (n = 3). *P < 0.05, **P < 0.01, and ***P < 0.001 versus vehicle treatment only (CTGF 0 ng/ml).

CTGF is a secreted protein, which can be released into extracellular space and can act as an autacoid [11]. To explore the possible role of CTGF in TGFβ1-induced collagen production, recombinant CTGF was used as a stimulator to add to HESCs. The collagen and HSP47 expression induced by CTGF was then investigated through Western blotting. Fig 4 shows that a significant increase in collagen and HSP47 expression was observed by CTGF stimulation, suggesting that TGFβ1-induced collagen production may be mediated by CTGF and/or HSP47 induction.

### TGFβ1 signaling for collagen and CTGF expression

To further study the mechanism of action involved in collagen induction by TGFβ1, pharmacological interventions were used to target some cellular signaling pathways downstream TGFβ receptor (TGFβR) and their effects were determined by Western blotting. These pharmacological inhibitors were PD98059 (PD) for MAPKK, SP600125 (SP) for JNK, SB202190 (SB) for p38 MAPK, LY294002 (LY) for PI-3K/Akt, actinomycin D (act. D) for DNA transcription, cycloheximide (CHX) for protein translation, and SIS3 for Smad2/3. The concentrations chosen were based on the observations that they could inhibit cellular signaling in several studies by our laboratory.

**Fig 5:**
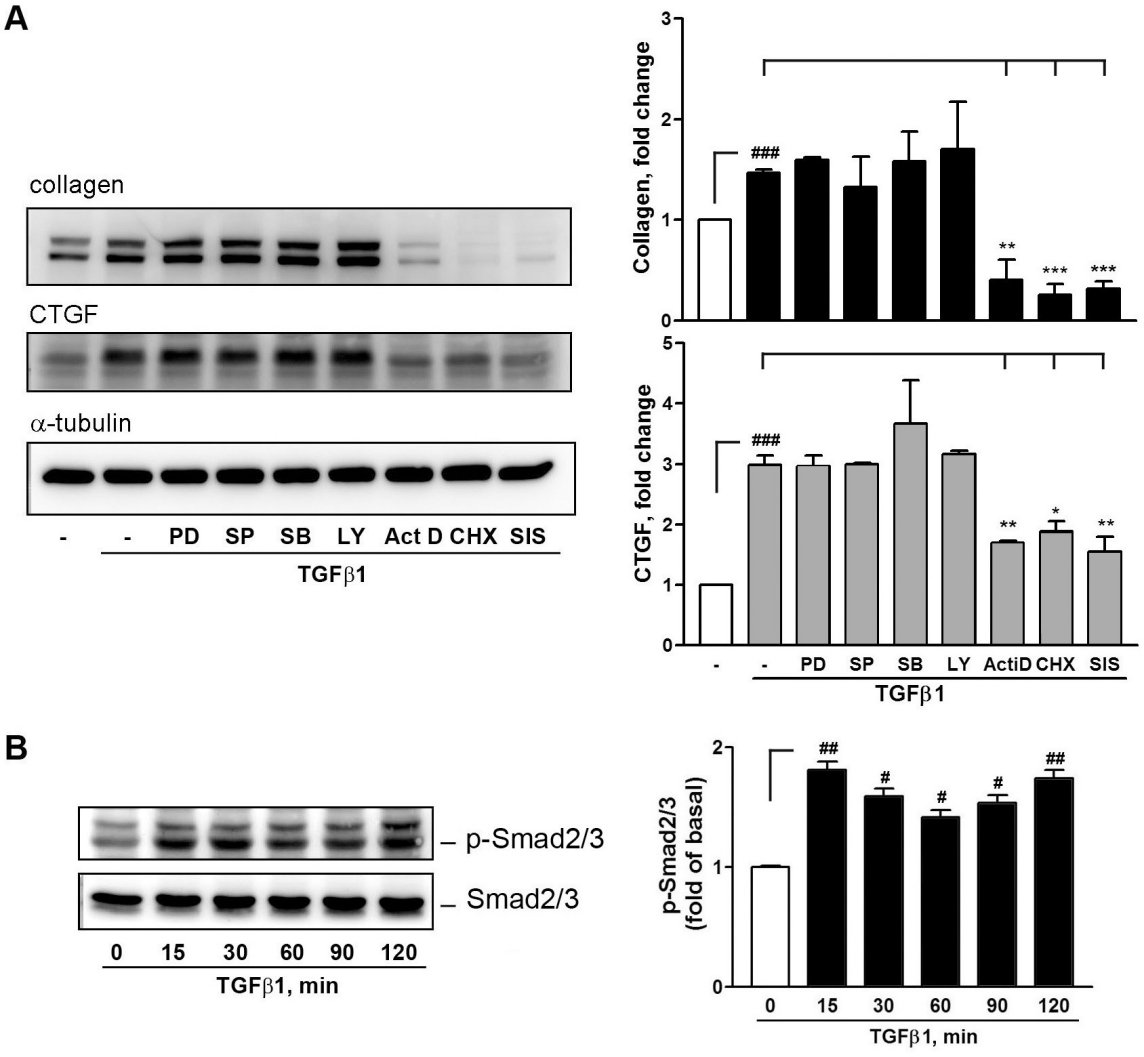
Effects of pharmacological interventions on TGFβ1 signaling in HESCs. (A) Measurement of pharmacological inhibitors on TGFβ1 signaling. Cells were pretreated with the indicated inhibitors for 30 min and followed by treatment with vehicle (basal) or TGFβ1 (5 ng/ml) for 16 h. The protein expression was analyzed through Western blotting (n = 3). The concentration for each inhibitor used is PD98059 (PD), SP600125 (SP), SB202190 (SB) (10 μM each), LY294002 (LY) (10 μM), actinomycin D (act. D, 1 μM), cycloheximide (2 μM) and SIS3 (10 μM). (B) TGFβ1 induced Smad2/3 activation. Cells were stimulated with TGFβ1 (5 ng/ml) for 0~120 min. The Smad2/3 expression and phosphorylation was analyzed through Western blotting and densitometry (n = 3). ^##^*P* < 0.01 and ^###^*P* < 0.001 versus basal level (vehicle treatment only,—or TGFβ1 0 ng/ml). *P < 0.05, **P < 0.01, and ***P < 0.001 versus control (TGFβ1 treatment only, -).

In Fig 5A, among these inhibitors, act. D, CHX, and SIS3 were found to inhibit TGFβ1-induced collagen and CTGF protein expression. Since SIS3 could inhibit Smad2/3, whether TGFβ1 directly affects Smad2/3 expression was measured through Western blotting. In Fig 5B, TGFβ1 significantly induced Smad2/3 phosphorylation. The induction appeared to be activated in a two-phase manner and the corresponding total Smad2/3 was not affected, suggesting that TGFβ1 affects collagen and CTGF expression through transcriptional regulation, possibly involving Smad2/3-related signaling pathway.

### siRNA knockdown (KD) of CTGF expression compromises TGFβ1-induced collagen expression

**Fig 6:**
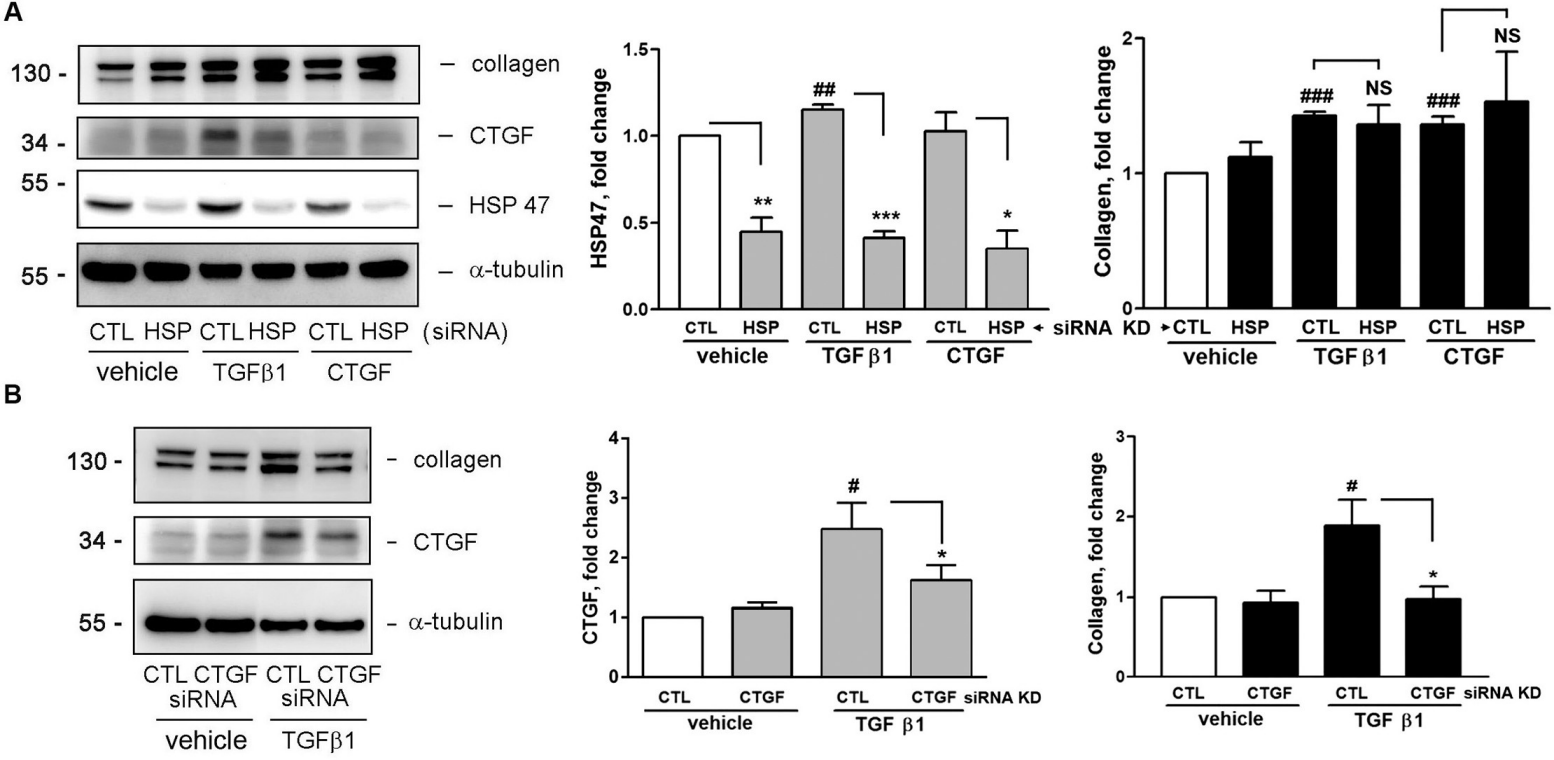
Effect of CTGF and HSP47 KD on TGFβ1-induced collagen expression in HESCs. (A and B) Cells transfected with control (CTL), CTGF, or HSP47 (HSP) siRNA were treated with vehicle, TGFβ1 (5 ng/ml), or CTGF (30 ng/ml) for 16 h. The protein expression was determined through Western blotting and quantified by densitometry (n = 3). ^*#*^*P* < 0.05, ^##^*P* < 0.01, and ^###^*P* < 0.001 versus control siRNA transfection with vehicle treatment (open bar). *P < 0.05, **P < 0.01, and ***P < 0.001 versus control (CTL). NS: non-significant.

We have shown that addition of CTGF to HESCs can lead to collagen and HSP47 expression (Fig 4). To explore whether CTGF and/or HSP47 are (is) involved in TGFβ1-induced collagen, small interfering RNAs (siRNAs) were used to KD CTGF and HSP47 expression, followed by examining collagen expression by TGFβ1 treatment. Fig 6 depicts that transfection of CTGF and HSP47 siRNA to the cells significantly reduced the expression of CTGF and HSP47, indicating both of the siRNAs worked efficiently on the HESCs. More importantly, HSP47 siRNA KD exhibited no inhibitory effect on TGFβ1- and CTGF-induced collagen expression. On the contrary, CTGF siRNA KD significantly abolished TGFβ1-induced collagen expression (Fig 6A and 6B). This reveals that TGFβ1-induced collagen expression through CTGF but not HSP47 expression

### Expression patterns of stromal (myo)fibroblast, TGFβ1, CTGF, and collagen in human endometrium specimens with adenomyosis

**Fig 7:**
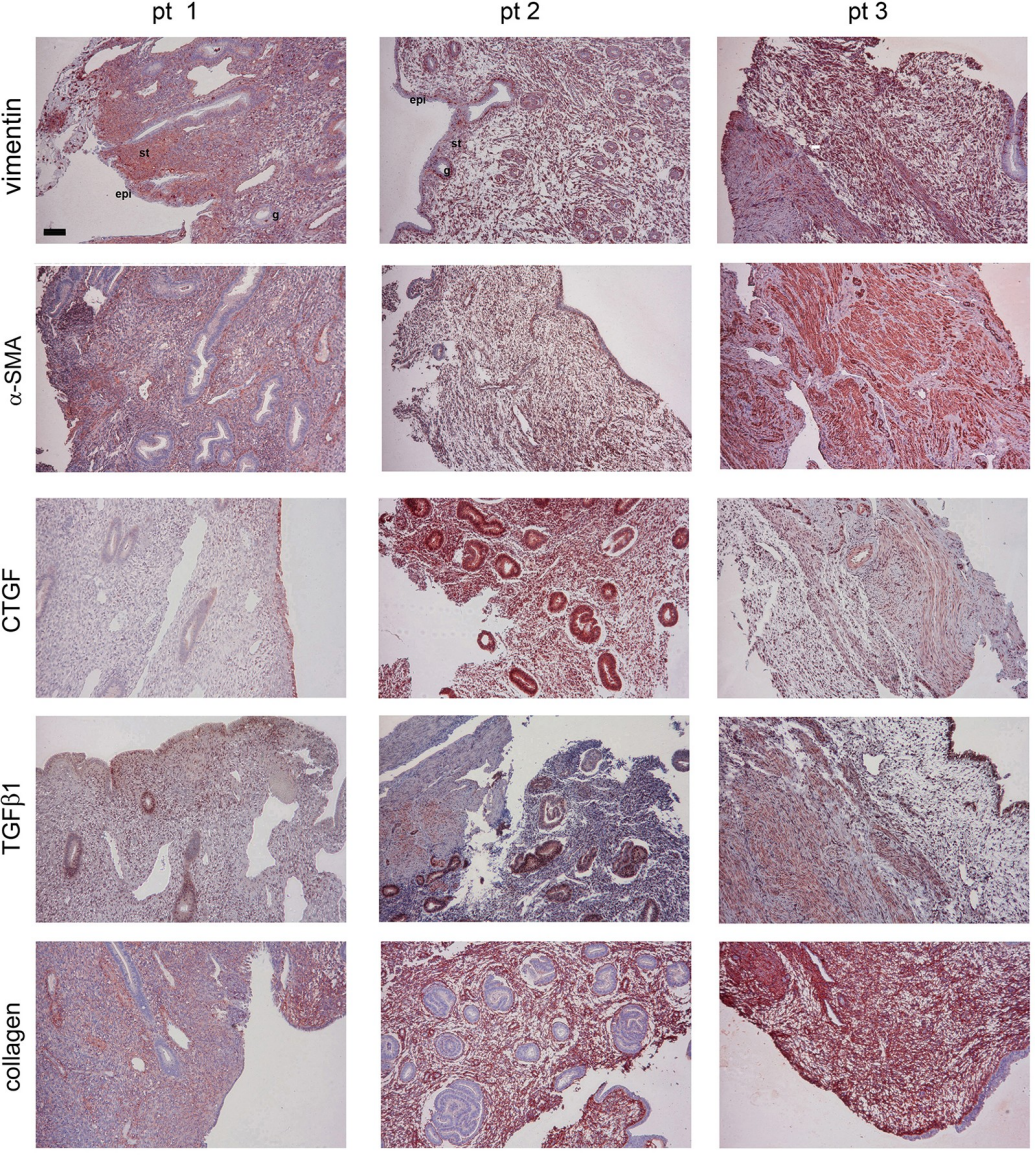
IHC of the expression patterns of (myo)fibroblast, TGFβ1, CTGF, and collagen in human adenomyotic endometrium specimens. The endometrium specimens from three individual patients (pts) were analyzed by IHC using the Abs raised against the indicated proteins. This was representative from three to four IHC results. Scale bar = 100 μm.

Next, IHC was performed to examine the (myo)fibroblast, CTGF, TGFβ1, and collagen expression patterns in the endometrium specimens with adenomyosis. Fig 7 shows a positive staining in vimentin and α-SMA in the region of subepithelial stroma of these tissues. Among the three patients, the α-SMA positive-staining region was approximately estimated to be 25% in each specimen of patient 1 and 2 and 75% in the specimen of patient 3, indicating stromal (myo)fibroblast expression in this region. Interestingly, CTGF was not only expressed in the stroma region but also in the epithelium and glands of endometrium. In parallel, collagen and TGFβ1 were also expressed in the stroma region of adenomyotic endometria.

## Discussion

An adenomyoma is a focal region of adenomyosis, which is a mass within uterine myometrium and is usually fibrotic. Although the exact etiology of adenomyosis is still elusive, all types of adenomyosis usually have a feature of fibrosis [2]. As described earlier, fibrosis is characterized by an excessive collagen and ECM deposition [26]. In this study, we found that TGFβ1 could induce collagen and CTGF expression in the HESCs (Fig 1B). IHC of the adenomyotic endometria demonstrates TGFβ1 and collagen expression in the endometrial subepithelial stroma (Fig 7), suggesting that they may be coexpressed in the pathological conditions. Interestingly, the inductory effect on collagen expression by TGFβ1 stimulation was not through directly affecting collagen gene expression but was mainly mediated via CTGF mRNA and protein expression. Although a previous study has shown that TGFβ1 can induce collagen, CTGF, α-SMA and fibronectin mRNA expression in endometriotic and endometrial stromal cells [27], this is the first time to elucidate the action mechanism that TGFβ1 is capable of inducing collagen expression mediated through induction of CTGF expression and cellular Smad2/3-dependent pathway in human adenomyotic endometrium-derived stromal fibroblasts. In addition, exogenous CTGF has been shown to induce collagen expression in human lung fibroblasts and CTGF participates in bleomycin-induced collagen expression and lung fibrosis [28, 29],

The following lines of evidence demonstrate how TGFβ1 induces collagen expression in HESCs through CTGF induction. First, we showed that TGFβ1 could simultaneously cause collagen and CTGF expression (Figs 1 and 2). Second, TGFβ1 did not affect collagen mRNA expression; however, it caused an increase in CTGF mRNA level. Third, addition of recombinant CTGF to HESCs led an increase in collagen expression in the stromal fibroblasts (Fig 4). Fourth, siRNA KD of CTGF mRNA to downregulate its protein expression comprised TGFβ1-induced collagen expression (Fig 6). All of these revealed an important role of CTGF in this induction process. CTGF has been recognized as an important player in fibrogenic pathways both in nonhepatic tissues and liver fibrosis [30]. It is also taken as a target of clinical trials in fibrotic liver diseases as well as a potential therapeutic target for cardiac fibrosis. For instance, angiotensin II is a well-known fibrotic factor for cardiac hypertrophy and fibrosis and can directly induce TGFβ and CCN2/CTGF [31]. Under that condition, CTGF works with TGFβ in a concerted way to induce cardiac fibrosis, in which TGFβ1 acts as a main inducer for triggering cellular signaling, whereas CTGF as a mediator for fibrogenesis. [32–34]. This phenomenon appears to be similar to those observed in this study. Taken together, we demonstrated here that CTGF is a critical and required mediator for TGFβ1-induced collagen expression in HESCs.

In Fig 2, we showed that TGFβ2 and 3 exhibited similar but relatively weak activity toward collagen and CTGF expression than TGFβ1 did (Fig 2B). TGFβs can induce cellular signaling through Smad-dependent and -independent pathways by activating distinct combinations of type TGFβ I and II receptors [35]. The canonical signaling pathway for TGFβs involves the cellular Smad family [36], whereas the noncanonical pathway involves MAPK and other signaling pathways [37]. Our results show that the inhibitors targeting PI-3K/Akt and MAPKs such as ERK1/2, p38 MAPK, and JNK failed to inhibit TGFβ1-induced collagen and CTGF expression, suggesting that TGFβ1 induces collagen expression through a canonical fashion. Indeed, the collagen and CTGF expression could be reduced by SIS3, an inhibitor toward Smad2/3. In addition, actinomycin D and cycloheximide almost completely inhibited TGFβ1-induced collagen and CTGF expression. In accordance with these observations, TGFβ1 simultaneously induced Smad2/3 phosphorylation in HESCs (Fig 5). Therefore, our findings indicate that TGFβ1-induced collagen and CTGF expression through transcriptional and translational regulation, which involves Smad2/3-dependent pathways. As discussed earlier, TGFβ plays a central role in cardiac and liver fibrosis [30, 31]. In addition, TGFβ-induced α-SMA synthesis requires Smad3 in fibroblast activation [38]. Our IHC result also demonstrated that positive staining for (myo)fibroblast’s marker-vimentin and α-SMA, TGFβ1, collagen, and CTGF in the subepithelial stroma region of the human adenomyotic endometria (Fig 7), indicating that (myo)fibroblasts and these molecules may be colocalizedly expressed at the same region.

It has been shown that TGFβ1 drives EMT and FMT in adenomyosis development both in animal model and in human [15, 21], although the expression of TGFβ receptor type I to III and CTGF was found to be indifferent at EMJZ in adenomyosis uteri [20]. In this study, we showed that α-SMA was expressed in endometrium tissue, suggesting the presence of (myo)fibroblasts at this region. Although the expression of TGFβR and CTGF was found to be indifferent at EMJZ in adenomyosis uteri, higher expression of TGFβR ligand such as TGFβ1 may act as an important player in inducing adenomyosis.

**Fig 8:**
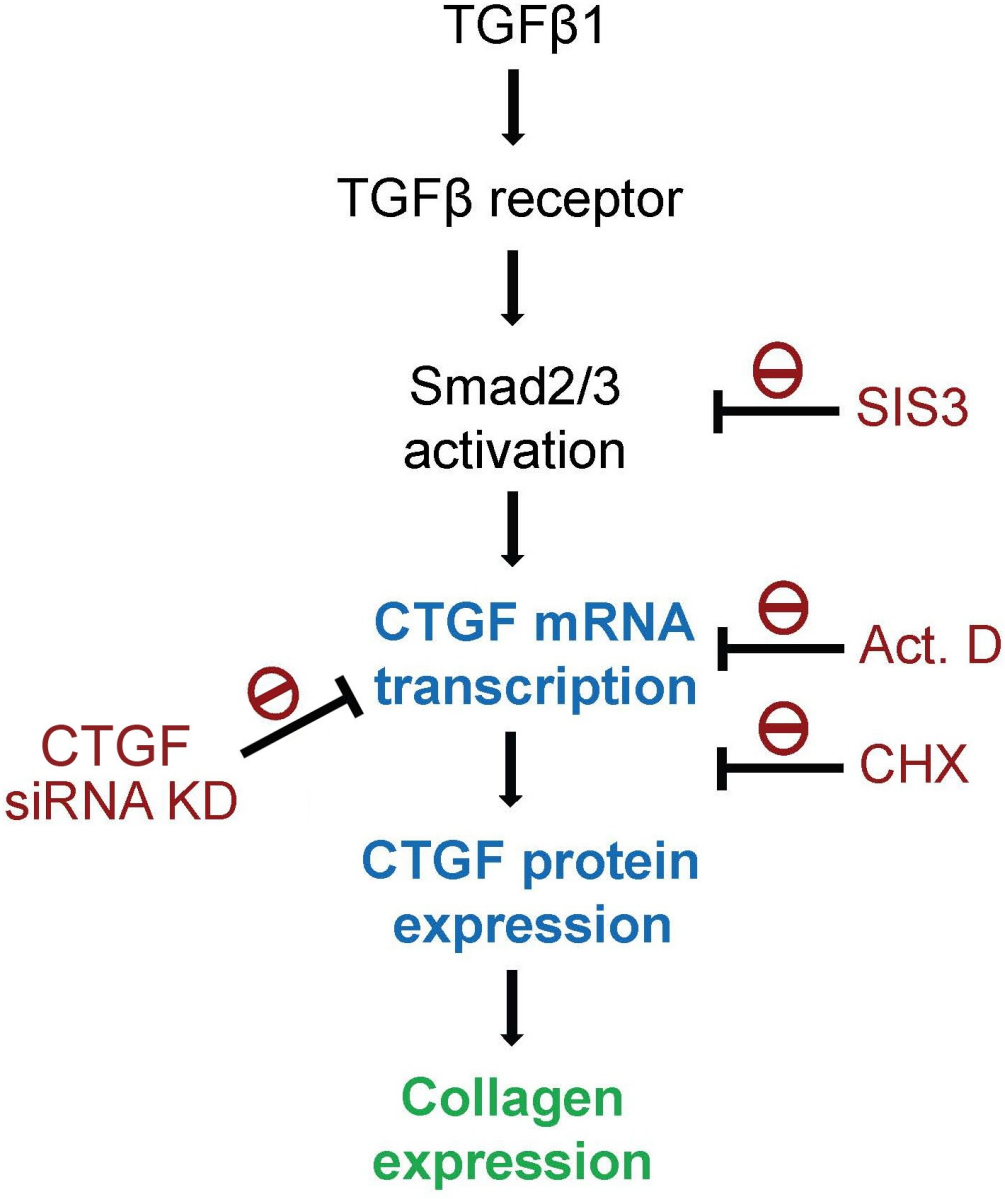
Proposed scheme of TGFβ1 signaling in inducing CTGF and collagen expression in human endometrial stromal cells.

In conclusion, we demonstrate here that TGFβ1, CTGF, and collagen are expressed in subepithelial stroma region of the endometrium with adenomyosis, in which it is abundant of fibroblasts with positive vimentin staining. TGFβ1 can upregulate collagen expression through CTGF induction in human endometrial stromal cells (fibroblasts). The possible mechanism for TGFβ1-induced collagen expression is presented in Fig 8. We provide evidence that endometrial TGFβ1 may cause fibrosis in endometrium and ectopic endometrium may participate in uterine adenomyosis.

## Supporting information

S1 Data The raw data for each Western blot result. (RAR)

## Abbreviations

CTGF: connective tissue growth factor
ECM: extracellular matrix
EMJZ: endometrial-myometrial junctional zone
HESC: human endometrial stromal cell
HSP47: heat shock protein 47
TGFβ: transforming growth factor β.
